# Unraveling the role of probiotics in affecting the structure of monoglyceride gelled emulsions: A low-field ^1^H NMR study

**DOI:** 10.1016/j.crfs.2024.100724

**Published:** 2024-03-28

**Authors:** Sofia Melchior, Eleonora Carini, Marcello Gigliotti, Francesco Ciuffarin, Marilena Marino, Nadia Innocente, Maria Cristina Nicoli, Sonia Calligaris

**Affiliations:** a Department of Agricultural, Food, Environmental and Animal Sciences, University of Udine, Via Sondrio 2/a, Udine, Italy; b Department of Food and Drug, University of Parma, Parco Area delle Scienze, 47/A, Parma, Italy

**Keywords:** *Lacticaseibacillus rhamnosus*, Rheology, ^1^H NMR analysis, Viability, Monoglyceride-structured emulsions

## Abstract

The capacity of monoglyceride (MG) gelled emulsions (MEs) in protecting probiotic cells of *Lacticaseibacillus rhamnosus* against stresses suffered during food processing, storage, and human digestion has been recently demonstrated. These findings open new perspectives on the possible participation of probiotics in the stabilization of emulsion structure. To unravel this aspect, rheological analysis and Low-Field ^1^H NMR investigations were performed on MEs having different aqueous phases (water or skimmed milk) and stored for increasing time (1 and 14 days) at 4 °C. Loaded and unloaded samples were considered. Results highlighted that probiotics initially hindered the ability of MG to self-assemble in the multiphase environment, interacting in some way with MG crystalline lamellar structure, as confirmed by rheological and ^1^H NMR analysis. During storage, an increase of proton compartmentation was observed in loaded MEs indicating the role of probiotics in stabilizing MG structure at a molecular level. Such a result was more evident when the system was composed of milk, suggesting that the presence of milk-native components (*i.e.,* lactose, proteins, and minerals) favored the cell-structure interactions. Such preliminary results could open new perspectives in considering probiotic cells as having an active role in the stabilization of food structure.

## Highlights

• The role of live probiotics was investigated in monoglyceride-structured emulsions.
• Probiotics initially hindered the formation of the monoglyceride (MG) network.
• Upon storage, cells had stabilized MG structure as confirmed by Low-Field ^1^H NMR.

## Graphical abstract

**Figure:**
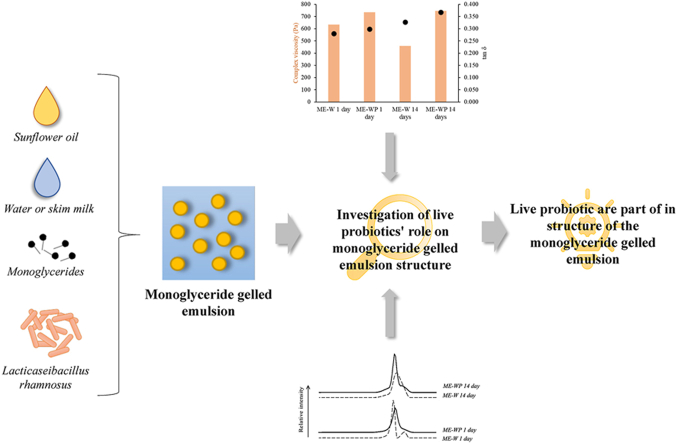

## 1 Introduction

Probiotic microorganisms are today under the spotlight due to their well-recognized health benefits, which are strictly associated with cell viability. Recent studies report positive health effects from inactivated microbial cells, in addition to living ones (Akter et al., 2020). Viable cells should be higher than 10^6^–10^7^ CFU/g for a food to be claimed as a probiotic (Akhtar et al., 2021). Despite the increasing number of functional products on the market, the inclusion of probiotics into foods is challenging due to several stresses that may compromise cell survival during the entire product life (Liu et al., 2019; Marino et al., 2021). To tackle these deadly conditions, several strategies have been proposed and encapsulation is one of the most promising to deliver probiotics and maintain their viability during food processing, storage, and gastrointestinal transit (Reque and Brandelli, 2021). Although great efforts were made to properly design delivery systems suitable for being incorporated into the food matrix while protecting cells, the structure/function relationships remain underexplored.

In non-food applications (*i.e.*, wastewater treatment, petroleum production, and bioremediation of aquifers) bacteria, yeasts, and viruses proved to stabilize foams and emulsions behaving as fine solid particles at interfaces. Thanks to their affinity for one another, bacteria with hydrophobic membranes self-assemble at the interface, providing better resistance to coalescence and deformation (Dorobantu et al., 2004; Heard et al., 2008; Jiang et al., 2021; Lahtinen et al., 2007). Recent studies used microorganisms as stabilizers also in food structures. Firoozmand and Rousseau (2016) demonstrated the ability of thermally inactivated baker’s yeast and lactic acid bacteria to act as Pickering-type particles to generate and stabilize oil-in-water emulsions. Yet, Jiang et al. (2019, 2021) proposed the surface engineering of bacteria as a possible strategy to create new structural building blocks. These authors successfully modified *Lactobacillus acidophilus* and *Lacticaseibacillus rhamnosus* using lauroyl chloride or octenyl succinic anhydride to increase the hydrophobic nature of cell walls thus better stabilizing the interface of foams and emulsions. The ability to stabilize emulsions through Pickering-related mechanisms has been demonstrated in *Bacillus* and *Saccharomyces*, among other microorganisms (Nejadmansouri et al., 2023). However, most of these studies exploited inactivated bacteria and when probiotic cultures were used their viability and metabolically active state were only marginally considered. Based on these considerations, in the present study, an additional piece of the story is tentatively inserted in this puzzle to provide insight into the possible role of viable cells of probiotic *L. rhamnosus* as a structuring agent in monoglyceride (MG) gelled emulsions (MEs). These systems were recently shown to protect bacteria during processing, storage, and *in vitro* digestion in both model systems and food prototypes (*i.e.,* Ricotta cheese and ice cream) (Calligaris et al., 2018; Marino et al., 2017; Melchior et al., 2021, 2022). It is well known that, in multiphase systems, saturated MG forms crystalline bilayers that lead, under defined physicochemical conditions, to the formation of tridimensional networks resulting in self-standing emulsion (Batte et al., 2007a, 2007b; Valoppi et al., 2015). The considered MSEs were widely characterized in previous studies for their structure at different lengths of scales (Batte et al., 2007a, 2007b; Valoppi et al., 2015). The stability of the emulsion is strictly linked to the swelling capacity of the lamellar phase and can be improved by using a charged group on the surface of the lipid bilayers to increase the repulsive forces and thus the swelling of MG structures in water (Krog and Sparsø, 2004). The presence of other molecules, such as milk components, greatly affected the lamellar phase structure (Melchior et al., 2021; Valoppi et al., 2015). Microbial cells, in this complex structure, place themselves prevalently in the aqueous domain near MG crystalline structures as recently demonstrated (Melchior et al., 2021). The open question is whether live bacteria could interact with the MG network impacting the system structure. The Low-Field ^1^H NMR (LF ^1^H NMR) technique was applied to unravel this point. This rapid and non-invasive technique is widely used to study food quality and stability throughout the investigation of ^1^H molecular dynamics and mobility. LF ^1^H NMR can distinguish protons bound in free water or more structured water or even protons attached to different solid components (e.g., lipids, proteins, and carbohydrates) (Parenti et al., 2021). LF ^1^H NMR was found successful in monitoring the ability of hydrocolloids to stabilize emulsions and hydrogels (Develioglu et al., 2020; Wang et al., 2021), the relationship between water mobility and the physical structure of MG stabilizing O/W emulsions (Goldstein et al., 2012), and the chemical and structural changes linked to thermal oxidation of PUFA’s rich emulsion (Resende et al., 2021). Therefore, in this study, the LF ^1^H NMR technique in combination with rheological measurements was applied to highlight the possible role of live probiotic bacteria in the structuring of MG-gelled emulsions made of different aqueous phases (water or skimmed milk) and stored at 4 °C for up to 14 days.

## 2 Materials and methods

### 2.1 Materials

Stearic acid and palmitic acid were purchased from Sigma Aldrich (Milano, Italy); saturated monoglycerides (MG) were provided by Kerry Ingredients and Flavour (Bristol, United Kingdom) (fatty acid composition: 1.4% C14:0, 59.8% C16:0, and 38.8% C18:0; melting point: 68.05 ± 0.5 °C). Maximum Recovery Diluent (MRD), MRS agar, MRS broth, and phosphate-buffered saline (PBS) were purchased from Oxoid (Milan, Italy). *L. rhamnosus* (Lyofast LRB) was purchased from Sacco Srl (Cadorago, Como, Italy). Species identification was confirmed by partial 16S rRNA gene amplification (Martino et al., 2013). Sunflower oil (SO) and UHT skimmed milk (proteins: 3.4%; carbohydrates: 5.1%; fat: 0.05%; salt: 0.10%; pH = 6.70) were purchased from a local market. Deionized water (Millipore S. A.S, Molsheim, France) was used.

### 2.2 Culture preparation

Overnight cultures of *L. rhamnosus* were prepared by sub-culturing 100 μL of stock cultures in 100 mL of MRS broth at 37 °C for 18 h under anaerobic conditions in anaerobic jars with a gas generating system (Oxoid). The cells were then recovered by centrifugation at 13,000×*g* for 10 min at 4 °C, washed three times, and resuspended in sterile PBS to a final viability of about 10^9^ CFU/mL.

### 2.3 Sample preparation

MEs with and without probiotic cells were prepared according to Marino et al. (2017). The lipid phase was composed by 36.4% (w/w) of SO and 7.2% (w/w) of cosurfactant–MG mixture made by MG and palmitic and stearic acid in a ratio of 5:1:1 (w:w:w), while the aqueous phase was composed by 56.4% (w/w) of water at pH 10.9 or UHT skimmed milk. Based on their composition, samples were referred to as ME-W and ME-M when the aqueous phase consisted of water and milk, respectively. Both aqueous and lipid phases were heated at 70 °C in a water bath until MG melting. Then, the two phases were mixed and homogenized with an Ultra-Turrax® T18 (IKA, Milan, Italy) at 1000×*g* for 20 s. Before mixing and homogenization, 1 mL of *L. rhamnosus* suspension was added to the samples containing probiotics resulting in ME-WP and ME-MP (final viability of about 10^7^ CFU/g). The samples were cooled in an ice bath and then placed in sterile airtight containers. All systems were prepared in two biological replicates and stored at 4 °C for up to 14 days.

### 2.4 pH measurement

The pH was measured using a standard pH meter (Hanna Instruments pH 301, Padua, Italy). All measurements were performed in duplicate at 25 °C.

### 2.5 Evaluation of the probiotic viability during storage

Aliquots (about 1 g) of each gel were suspended in 9 mL of MRD and homogenized for 2 min. Decimal dilutions in MRD were then spread-plated onto MRS agar and incubated anaerobically at 37 °C for 48 h. Analysis was carried out after 1 and 14 days of storage at 4 °C.

### 2.6 Rheological properties

Rheological properties of samples were determined at 20 °C with a Haake Rheostress 6000 (40 mm parallel-plate geometry; 2 mm gap) (Thermo Scientific, Rheostress, Haake, Germany), equipped with the software Haake Rheowin v.4.60.0001 (Thermo Scientific). The stress sweep test was carried out at 1 Hz in the 0.1–100 Pa range. The frequency sweep test was performed by increasing the frequency from 0.1 to 10 Hz using a fixed stress value included in the linear viscoelastic region. Loss tangent (tan δ = G’’/G′) and complex viscosity (η* = [(G′)^2^ +(G″)^2^]^0.5^/ω) were obtained.

### 2.7 Low Field ^1^H NMR

A low resolution (20 MHz) ^1^H NMR spectrometer (the Minispec, Bruker Biospin, Milan, Italy) operating at 25.0 ± 0.1 °C was used to study the proton molecular mobility of systems. Almost 2 g of sample were placed into an NMR tube (10 mm diameter), that was sealed with Parafilm® to avoid moisture loss during the NMR experiment. The Carr-Purcell-Meiboom-Gill (CPMG) sequence was used to measure the spin-spin relaxation time (^1^H T_2_). The parameters used in the CPMG pulse sequence were as follows: a recycle delay of 10 s, an interpulse spacing of 0.02 ms, and 20,000 data points. ^1^H T_2_ curves were analyzed as quasi-continuous distributions of relaxation times (UPENWin software v. 1.04, Alma Mater Studiorum, Bologna, Italy). Default values for all UPEN parameters were used except for one parameter (LoXtrap) that was set to 1 to avoid extrapolation of relaxation times shorter than the first experimental point. Two tubes were analyzed for each sample batch (2) acquiring five CPMG curves for each tube, for a total of 20 experimental curves for each sample.

### 2.8 Statistical analysis

All results were expressed as the mean ± standard deviation (SD) of at least three measurements from two experimental replications. The *t*-test was performed using R v. 4.3.2 for Windows (the R foundation for statistical computing).

## 3 Results and discussion

### 3.1 Rheological properties of ME samples

Table 1 reports tan δ and complex viscosity of the MEs containing 10^7^ CFU/g of microbial cells. Unloaded samples were also studied as controls. All samples were characterized by rheological parameters typical of a gel-like behaviour, in agreement with previous literature reports (Melchior et al., 2021). ME-W was characterized by tan δ higher than that observed for ME-M while complex viscosity and lower. It should be noted that the pH of the systems was in the range between 5.0 and 5.6 and was maintained during the storage time, in agreement with previous observations (Melchior et al., 2021). After 14 days at 4 °C, a significant increase (*p* < 0.05) in complex viscosity was observed probably indicating the formation of stronger interactions among MG lamellas upon storage. The same trend was observed in ME-M. However, in this case independently of storage time, tan δ was higher while the complex viscosity was lower, respectively for ME-W and ME-M. These results confirmed the literature well describing the interfering effect on the MG structure of native milk components (*i.e.,* lactose, proteins, and minerals) (Melchior et al., 2021; Valoppi et al., 2015). Interestingly, just after preparation, lower complex viscosity was detected in both systems containing probiotics in comparison to unloaded samples. The addition of live cells probably made the system more crowded complicating the network formation. Lactic acid bacteria may be using monoglycerides as a source of energy, which could be the reason for the occurrence. This might initially disrupt the system and hinder the self-organization of monoglycerides. In support of this hypothesis, it should be noted that LAB possess enzymes such as lipases and esterases that enable them to hydrolyze lipids, including monoglycerides, into glycerol and fatty acids (Katz et al., 2002).

**Table 1:** Tan δ and complex viscosity of monoglyceride structured emulsions (ME) where the aqueous phase was water (ME-W) or milk (ME-M). ME-WP and ME-MP are the relevant samples added with probiotic bacteria.

Sample	Storage time (days)	tan δ (−)	Complex viscosity (Pa)	Viability (log CFU/g)
ME-W	1	0.280 ± 0.007 ^b,A^	632.7 ± 28.1 ^a,B^	–
	14	0.299 ± 0.010 ^b,A^	735.8 ± 51.0 ^a,A^	–
ME-WP	1	0.327 ± 0.022 ^a,A^	459.6 ± 99.0 ^b,B^	7.35 ± 0.02 ^A^
	14	0.367 ± 0.033 ^a,A^	747.0 ± 73.2 ^a,A^	7.36 ± 0.11 ^A^
ME-M	1	0.454 ± 0.003 ^a,A^	510.4 ± 25.9 ^a,B^	–
	14	0.474 ± 0.051 ^a,A^	698.4 ± 28.4 ^a,A^	–
ME-MP	1	0.440 ± 0.009 ^a,A^	293.7 ± 41.5 ^b,B^	7.18 ± 0.09 ^A^
	14	0.435 ± 0.010 ^a,A^	576.5 ± 42.5 ^b,A^	7.39 ± 0.08 ^A^

^a-b^ indicated a significant difference (*p* < 0.05) between samples with and without probiotics at same storage time and with the same composition.

^A−B^ indicated a significant difference (*p* < 0.05) between samples with the same composition at 1 and 14 days.

During storage, a significant increase in complex viscosity was noted in both loaded and unloaded ME-W and ME-M. The magnitude of the increase in complex viscosity was higher for loaded samples (+62.5 and + 96.3% for ME-WP and ME-MP, respectively) compared to the corresponding control samples (+16.3 and + 36.8% for ME-W and ME-M, respectively). According to our previous results, the protective capacity of the system to maintain probiotic cell viability during storage was confirmed (Marino et al., 2017; Melchior et al., 2021), since no changes in the microbial count were observed.

It can be inferred that probiotic bacteria exert different and opposite roles depending on the stage of ME storage and structure. The lower value of complex viscosity detected for probiotic-containing samples suggests that bacteria hinder the initial ability of MG to self-assemble in the multiphase system. However, along with storage, they appear to act as structuring building blocks as shown by the remarkable rise in complex viscosity detected after 14-day storage. In particular, due to the hydrophilic nature of the external part of the cell surface of Gram-positives, made mainly by peptidoglycan, *L. rhamnosus* presumably supports MG in the stabilization of aqueous phase structure contributing to the network reinforcement (Jiang et al., 2021). This hypothesis was also confirmed by the location of probiotic cells which was demonstrated to be mainly in the aqueous domain near MGs as observed by Melchior et al. (2021). In other words, live bacteria could act as Pickering stabilizing agents, in agreement with Firoozmand and Rousseau (2016), who demonstrated the ability of thermally-inactivated baker’s yeast and lactic acid bacteria to stabilize oil-in-water emulsions by colonizing the interface.

### 3.2 Low field ^1^H NMR relaxometry of ME samples

**Fig. 1:**
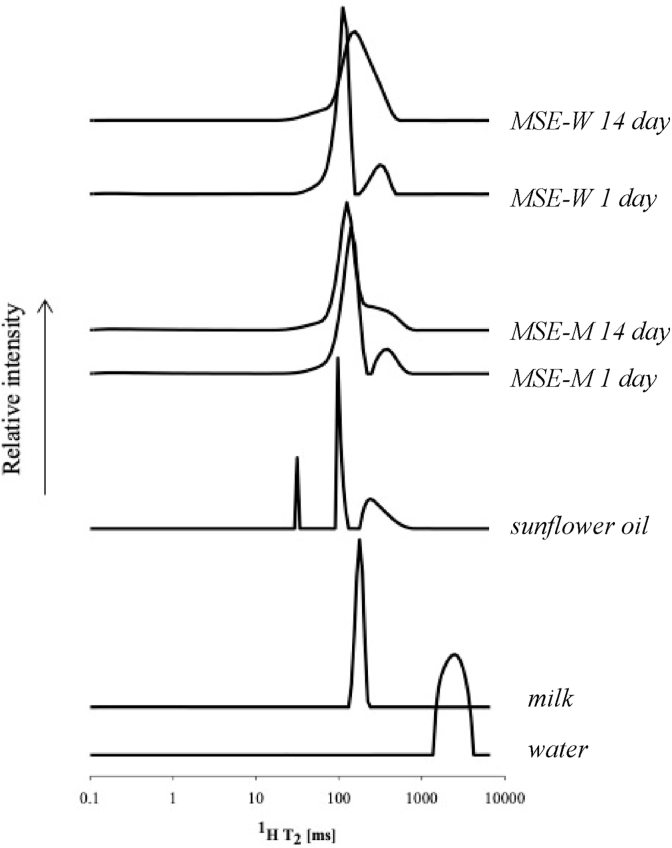
Representative ^1^H T_2_ distributions of relaxation times of water, milk, sunflower oil, and ME-W and ME-M at day 1 and after 14 days of storage at 4 °C.

To further investigate the potential role of probiotics as structuring agents, the Low Field ^1^H NMR technique was used. The representative ^1^H T_2_ distributions of relaxation times of ME-W and ME-M are reported in Fig. 1, while T_2_ relaxation times and relative abundances of proton populations can be found in Table 2. In order to better elucidate the effect of the composition on the ^1^H T_2_ mobility and dynamics in MEs, the CPMG of water, milk, and sunflower oil (SO) were analyzed as well (Fig. 1). Line shape of ^1^H T_2_ distributions of ME-W and ME-M were similar and depicted three well-resolved populations: one prevalent population at intermediate mobility (Pop B), one with shorter T_2_ (Pop A) representing a very small abundance of protons, and the third one (Pop C) at higher mobility (∼17% of total protons). This third population seemed to reflect the presence of oil as it showed relaxation in a similar time range as observed for SO and comparable to previously reported data (Goldstein et al., 2012; Wang et al., 2020). In particular, the protons of the more unsaturated fractions of SO were likely those observed in the Pop C detected in structured systems. In ME-W, the main proton population (Pop B) relaxed at ∼113 ms encompassing ∼82% of total protons while the less mobile population (Pop A) relaxed at ∼0.22 ms with a very low relative abundance (<1%). Two ^1^H T_2_ populations (∼0.43 ms and ∼184 ms) were found instead of three in structured gel composed of 55.2% canola oil, 40% water, and 4.5% saturated MG (Goldstein et al., 2012). The discrepancy between the findings of these authors and our data could be explained by the different types and amounts of ingredients used to prepare the emulsions and the different algorithms used to elaborate CMPG curves which may result in different absolute values of relaxation times. Although a similar behaviour was detected, a change in proton mobility was observed when the aqueous phase was changed from water to milk (ME-W vs ME-M) with higher mobility of Pop C due to the presence of milk (∼300 vs ∼358 ms, respectively). These results may be related to the weaker solid-like behaviour of ME-M as compared to ME-W (Table 1) confirming the critical role of the aqueous phase composition in ME structure. In particular, it has been previously demonstrated that the presence of native milk components displaced MG at the interface changing their structuring ability (Valoppi et al., 2015).

**Table 2:** ^1^H T_2_ and relative abundances of ^1^H populations for systems at day 1 and after 14 days of storage.

Sample	Storage time (days)	^1^H T_2_ (ms)			^1^H abundance (%)		
		Pop A	Pop B	Pop C	Pop A	Pop B	Pop C
Water	–	–	–	2655.00 ± 161.87	–	–	100.00 ± 0.00
Skim milk	–	–	–	217.41 ± 5.64	–	–	100.00 ± 0.00
Sunflower oil	–	63.49 ± 0.28	140.87 ± 2.80	322.58 ± 0.63	36.35 ± 0.59	32.74 ± 0.12	30.88 ± 0.72
ME-W	1	0.22 ± 0.03	112.90 ± 2.45	299.53 ± 14.40	0.73 ± 0.06	82.04 ± 0.82	17.23 ± 0.79
	14	0.27 ± 0.02	139.48 ± 10.99		1.15 ± 0.13	99.28 ± 0.60	
ME-WP	1	0.23 ± 0.03	137.66 ± 2.42	–	1.36 ± 0.23	98.64 ± 0.23	–
	14	0.24 ± 0.04	132.30 ± 4.45	276.02 ± 20.05	1.41 ± 0.19	83.49 ± 0.62	15.10 ± 0.51
ME-M	1	0.21 ± 0.02	138.23 ± 6.40	357.94 ± 20.56	2.21 ± 0.20	85.48 ± 7.63	16.40 ± 1.22
	14	0.21 ± 0.02	126.50 ± 4.24		2.07 ± 0.44	97.93 ± 0.44	
ME-MP	1	0.22 ± 0.01	115.77 ± 6.19	–	2.45 ± 0.22	97.55 ± 0.22	–
	14	0.21 ± 0.02	121.17 ± 6.78	323.90 ± 25.49	2.30 ± 0.15	80.42 ± 1.05	17.28 ± 0.97

After 14 days of storage, the well-resolved prevalent (Pop B) and more mobile (Pop C) populations were merged into a single broad population without mobility changes, suggesting an increase of the molecular environment homogeneity or, in other words, an overall decrease of protons compartmentation, as an effect of storage time. This implied a more extensive and effective exchange among protons of different domains. The increase in fast exchange could be a sign of a rearrangement occurring in the MG network. ME systems studied in the present work showed an increase of complex viscosity highlighting some structural changes occurring during storage, possibly driven also by a molecular rearrangement as detected by ^1^H T_2_ dynamics. Goldstein and co-authors (2012) studied the ^1^H T_2_ mobility in similar systems during a period of storage up to 28 days at 20 °C and 30 °C and no changes were detected in the mobility of the observed more mobile proton population, confirming our data.

**Fig. 2:**
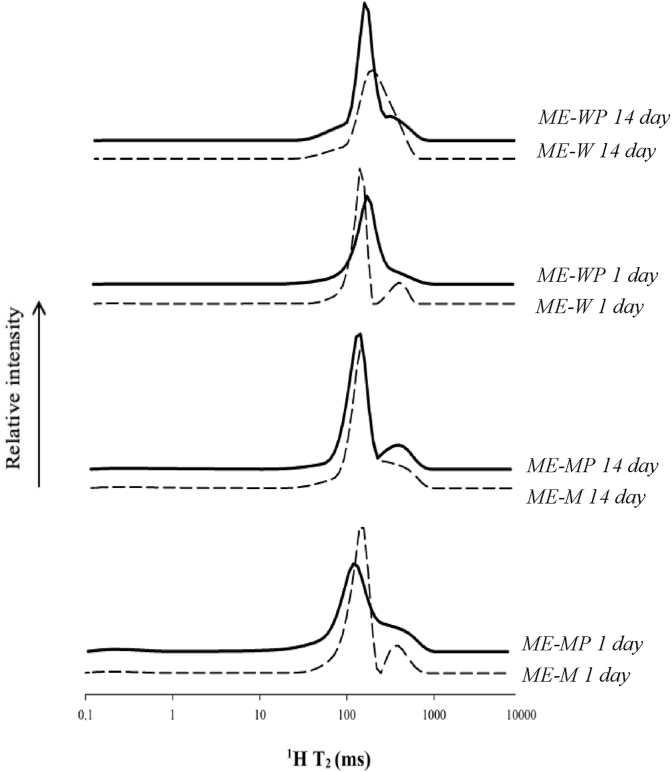
^1^H T_2_ distributions of relaxation times for ME with (**––––**) and without (**- - - -**) probiotics at day 1 and after 14 days of storage at 4 °C.

The inclusion of probiotic *L. rhamnosus* cells in MEs changed the ^1^H T_2_ dynamics and mobility (Fig. 2 and Table 2, day 1). Pop B and C were not still well resolved after probiotic inclusion in both ME-WP and ME-MP; moreover, the main population resulting from the merging of Pop B and Pop C peaked at shorter relaxation times in ME-MP if compared with ME-M. These findings confirmed a possible role of probiotics in the network structuring as previously observed and this role was more important when milk rather than water was the aqueous phase. The major change observed in the presence of milk may be related to the presence of a nutritious medium in this system, which may have favored some interactions between microorganisms and solutes (*i.e.*, lactose, proteins, and minerals). The location of probiotic cells, close to MG structures and protein aggregates, observed in other studies (Melchior et al., 2021, 2022), may strengthen this speculation.

Comparing samples, lower molecular mobility in ME-MP than ME-WP was found, despite other results (Table 1) and mesoscopic and macroscopic properties probed in previous work (Melchior et al., 2021) indicated that the replacement of water with milk reduced the strength of the gel network *i.e.*, decreased hardness and Storage Modulus (G’). This behavior was not so surprising but rather deserves interest and future investigation. Further changes were noticed during storage (Fig. 2 and Table 1). Storage did not change the molecular mobility but an increase of protons compartmentation (Pop B and C better resolved at day 14 than at day 1) in systems including probiotics was found. This was the opposite behaviour encountered in systems without probiotics where storage decreased protons compartmentation (PopB and PopC merged in a single broad population after 14 days). Thus, it seems that probiotics were able to hinder the network rearrangement during storage highlighting their possible stabilizing effect at a molecular level, possibly due to probiotics-network interactions developed, as hypothesized above.

## 4 Conclusions

This work represents a step forward in understanding the role of probiotic microorganisms in food structure. Results highlighted that live probiotic microorganisms contributed to the stabilization of monoglyceride-gelled emulsion during storage when the network rearrangement occurs. Such effect seems to be strictly dependent on the system composition, being more effective when nutritional compounds, such as proteins, lactose, and minerals, are present. Although other investigation is required, this preliminary work opens the possibility of exploiting live probiotic bacteria as novel and health-promoting structuring ingredients.

## CRediT authorship contribution statement

**Sofia Melchior:** Formal analysis, Investigation, Writing – original draft, Writing – review & editing, Visualization. **Eleonora Carini:** Conceptualization, Methodology, Formal analysis, Investigation, Resources, Writing – original draft, Writing – review & editing, Visualization, Supervision. **Marcello Gigliotti:** Formal analysis, Investigation, Writing – original draft, Writing – review & editing, Visualization. **Francesco Ciuffarin:** Formal analysis, Visualization. **Marilena Marino:** Formal analysis, Investigation, Resources, Writing – review & editing, Supervision. **Nadia Innocente:** Resources, Writing – review & editing, Supervision. **Maria Cristina Nicoli:** Resources, Writing – review & editing, Supervision. **Sonia Calligaris:** Conceptualization, Resources, Writing – review & editing, Visualization, Supervision.

## Declaration of competing interest

The authors declare that they have no known competing financial interests or personal relationships that could have appeared to influence the work reported in this paper.

## Data Availability

Data will be made available on request.
